# Methodological considerations for nonconcurrent comparisons of pulsed field ablation catheter platforms

**DOI:** 10.1016/j.hroo.2026.01.004

**Published:** 2026-01-13

**Authors:** Ankur Sharma, Varshini Vadhithala, Arun Kumar, Sushma Verma, Sushma Narsing Katkuri

**Affiliations:** 1 Department of Anatomy, School of Medical Sciences and Research, Sharda University, Greater Noida, Uttar Pradesh, India; 2 Dr. D. Y. Patil Medical College Hospital and Research Centre, Dr. D. Y. Patil Vidyapeeth (Deemed-to-be-University), Pimpri, Pune, Maharashtra, India; 3 Faculty of Pharmaceutical Sciences, Graphic Era Hill University, Dehradun, Uttarakhand, India; 4 Centre for Promotion of Research, Graphic Era (Deemed to be University), Dehradun, Uttarakhand, India; 5 Department of Pharmaceutics, Noida Institute of Engineering & Technology (Pharmacy Institute), Greater Noida, Uttar Pradesh, India; 6 Department of Community Medicine, Malla Reddy Institute of Medical Sciences, Malla Reddy Vishwavidyapeeth, Suraram, Hyderabad, Telangana, India

We read with interest the retrospective comparison of variable-loop vs fixed-loop circular catheters for pulmonary vein isolation using pulsed field ablation.^1^ The consecutive real-world experience and clear procedural descriptions are strengths that help readers judge feasibility and workflow.

Interpretation of comparative effects is constrained by design. The cohorts appear nonconcurrent, with catheter choice largely determined by calendar time. Nonconcurrent comparisons are vulnerable to secular changes in case mix, periprocedural management, mapping strategy, and adjunctive technology, which can introduce time-related confounding even after covariate adjustment.^2^^,^^3^ Operator and institutional learning may further bias early outcomes for a newly adopted platform; reporting procedure order and considering models that incorporate case order and calendar time can help distinguish device effects from learning effects.^4^ In addition, discretionary cointerventions, such as additional posterior wall or carina ablation and differing guidance approaches (fluoroscopy-only vs electroanatomic mapping), should be standardized or examined in sensitivity analyses, as they can influence procedure duration and acute end points. Clarifying the handling of missing data and any apparent discordance between summary statistics and baseline *P* values would further strengthen transparency.

The findings are informative but should be viewed as hypothesis generating until confirmed in contemporaneous comparative studies aligned to a target trial specification.

## References

[1] Yogarajah J., Hutter J., Kahle P. (2025). Real-world comparison of variable vs fixed-loop circular pulsed field ablation catheters: acute outcomes including non-pulmonary vein ablation. Heart Rhythm O2.

[2] Bofill Roig M., Burgwinkel C., Garczarek U. (2023). On the use of non-concurrent controls in platform trials: a scoping review. Trials.

[3] Matthews A.A., Danaei G., Islam N., Kurth T. (2022). Target trial emulation: applying principles of randomised trials to observational studies. BMJ.

[4] Ssemaganda H., Davis S., Govindarajulu U. (2025). A statistical framework to detect and quantify operator-learning curves in medical device safety evaluation. Med Devices (Auckl).

